# Eliciting CD4-mimicking broadly neutralizing antibodies: new avenues towards the rational design of an HIV vaccine

**DOI:** 10.1038/s41392-024-01776-6

**Published:** 2024-02-29

**Authors:** Frauke Muecksch, Oliver T. Fackler

**Affiliations:** 1 Department of Infectious Diseases, Heidelberg University, Medical Faculty Heidelberg, Virology, Center for Integrative Infectious Disease Research (CIID), Heidelberg, Germany; 2 Chica and Heinz Schaller (CHS) Research Group, Department of Infectious Diseases, Virology, University Hospital Heidelberg, Heidelberg, Germany; 3 Department of Infectious Diseases, Heidelberg University, Medical Faculty Heidelberg, Integrative Virology, Center for Integrative Infectious Disease Research (CIID), Heidelberg, Germany; 4 German Centre for Infection Research (DZIF), Partner Site Heidelberg, Heidelberg, Germany

**Keywords:** Vaccines, Vaccines

In a study recently published in *Cell*, Saunders et al. report that a new anti-HIV vaccine triggers antibody responses resembling CD4-binding site broadly neutralizing antibody precursors in rhesus macaques.^1^ This study provides important proof of concept and establishes a framework for the rational design of an effective HIV vaccine.

Developing safe and broadly acting vaccines to generate sterilizing immunity is the holy grail in infectious disease research. The recent advent of mRNA vaccines against SARS-CoV-2 infection marks the fastest development of an effective vaccine in the history of biomedical research. In contrast, despite decades of intense research, none of the many Human Immunodeficiency Virus (HIV) vaccine candidates has progressed to routine clinical application. While antiretroviral drugs can control virus replication and prevent disease progression, we still lack a preventive or therapeutic vaccine. Ideally, a vaccine should trigger all arms of immunity including innate immune responses to drive subsequent adaptive immunity, characterized by specific and effective cytotoxic T cells as well as neutralizing antibody-producing B cells. Mounting those antibody responses, particularly against HIV, poses a significant challenge as only a minority of individuals, so called elite neutralizers, develop potent humoral immunity. These difficulties reflect important barriers to antibody neutralization imposed by, e.g., HIV’s genetic variability, the complex architecture of the viral glycoprotein Env, limited accessibility of key neutralization epitopes and general impairment of CD4 T cell help and B cell function.^2^

**Fig. 1:**
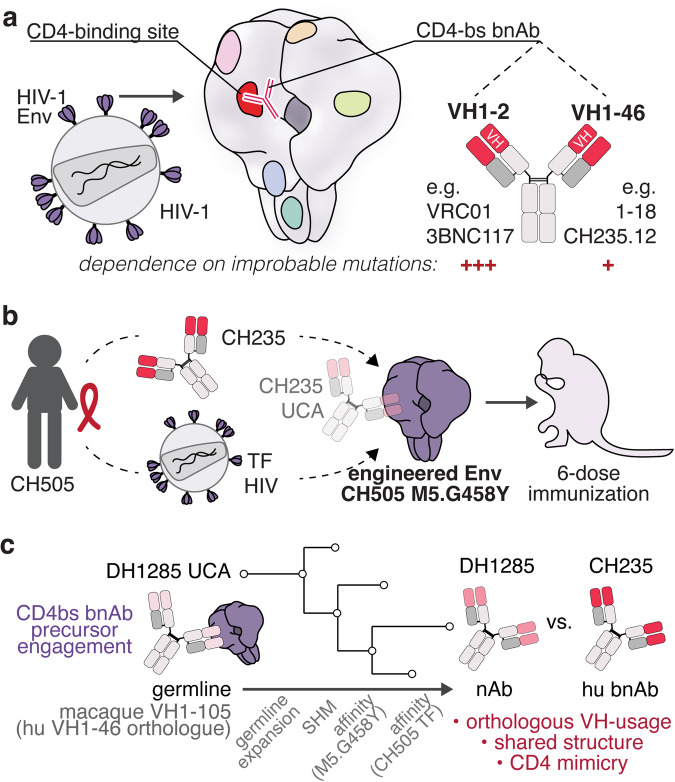
Elicitation of CD4-mimicking neutralizing antibodies by a novel HIV-1 vaccine. **a** Schematic illustration of the HIV-1 Envelope (Env) glycoprotein trimer with its conserved epitopes: membrane-proximal external region (MPER, green), gp120-gp41 interface (blue), gp120 “silent face” (light green), V1V2 Loop (orange), V3 loop (pink), CD4-binding site (CD4-bs, red). In red, examples for known CD4-bs broadly-neutralizing antibody (bnAb) of the variable heavy 1-2 (VH1-2) and VH1-46 class are shown. **b** Generation of the Env immunogen used in this study to immunize rhesus macaques. The CH505 M5.G458Y immunogen was derived from a transmitted founder (TF) virus isolated from participant CH505 and designed to bind to the CH235 bnAb universal common ancestor (UCA). **c** Immunogen binding to the putative DH1285 precursor antibody results in expansion of CD4bs-specific Abs with increasing affinity to the immunogen used for vaccination and emergence of TF Env recognition. This results in the neutralizing antibody (nAb) DH1285, which shares several features with the human bnAb CH235

Dissecting antibody responses of elite neutralizers led to the identification of several potent broadly neutralizing antibodies (bnAbs) that provide significant therapeutic benefit through passive immunization.^3^ Shaping humoral immunity towards bnAb generation in the context of protective vaccination, however, remains a major challenge. HIV bnAbs target conserved Env epitopes including the binding site for the HIV-receptor CD4 (CD4bs) (Fig. 1a). The CD4bs is an attractive vaccine target as many CD4bs bnAbs show high potency and breadth and provide protection via passive immunization in animal models and human clinical trials.^3^ Two types of CD4bs-targeting bnAbs have been identified: CD4-mimetic and HCDR3-binder bnAbs. CD4-mimetic bnAbs employ structural mimicry of the immune-globulin-like N-terminal domain of CD4 and are derived from closely related variable heavy 1-2 (VH1-2) or VH1-46 chain gene segments. However, since N-glycans sterically obstruct the Env-antibody interaction, the CD4bs is a challenging epitope for bnAb generation.

A promising vaccine approach to elicit potent HIV bnAbs is the specific activation of rare B cell bnAb precursors by germline-targeting. This strategy was born from the realization that germline forms of several HIV bnAbs cannot bind natively glycosylated Env and that engaging those rare germline B-cell receptors to initiate bnAb development requires engineering of suitable immunogens. Germline-targeting vaccine design has largely focused on VH1-2*02 class CD4bs bnAbs like VRC01 and 3BNC117 which are among the most potent and broadest HIV bnAbs. They contain a short five-residue light chain complementary determining region 3 (CDRL3) and a short and/or flexible CDRL1, allowing accommodation of the heavily glycosylated CD4bs. However, the requirement for many improbable mutations, insertions, and deletions to acquire full breadth represents a major bottleneck for VH1-2 class bnAb elicitation. In contrast, VH1-46-derived bnAbs such as CH235 and 1-18 do neither require the five-residue CDRL3 identified in VRC01-class antibodies nor insertions or deletions for acquisition of breadth.

To exploit these attractive properties of VH1-46-derived bnAbs, Saunders et al. extensively investigate memory B cell responses elicited by a VH1-46 germline-targeting vaccine based on the engineered CH505 M5.G458Y Env. The authors previously showed that this immunogen binds the universal common ancestor (UCA) Ab derived from VH1-46 of the CH235 bnAb lineage and induces CD4bs serum neutralizing Abs in macaques, but their precise quality was unknown.

To address whether this engineered immunogen can elicit VH1-46-derived CD4-mimetic bnAb precursors, three macaques received six doses of engineered M5.G458Y Env trimers with an adjuvant consisting of lipid nanoparticles to trigger innate immunity and robust generation of T follicular helper cells. Usage of lipid nanoparticles as adjuvants is based on the result that the adjuvant properties of lipid nanoparticle-based mRNA vaccines are at least part due to their nanoparticle formulation.^4^ Saunders and colleagues successfully transfer this approach to the administration of the HIV protein immunogen, collectively resulting in a serum CD4bs Ab response with CH235 UCA-like binding modes. To specifically characterize vaccine-induced Abs and compare them with known VH1-46 bnAbs, they isolated antibody sequences from memory B cells specific for M5.G458Y Env and expressed 53 sequences as recombinant IgG for functional analyses. 58% of all mAbs neutralized the vaccine-matched autologous virus, 68% of which exhibited CD4bs specificity similar to the CH235 lineage. Four of the antibodies competing with CH235 for Env binding were derived from the macaque gene IGHV1-105, the ortholog of human VH1-46. These showed high affinity for the vaccine immunogen and intermediate binding to the CH505 transmitted founder (TF) Env. Among these, DH1285 displayed the broadest neutralization across a panel of CH505 TF viruses, its potency closely resembling that of CH235-12. Structure determination by Cryo-EM confirmed that DH1285 binding to CH505 TF Env mirrors key structural signatures of the CD4-mimetic germline-derived HIV bnAbs derived from VH1-2 and VH1-46. DH1285 contains functional canonical germline-encoded amino acids present in known CD4-mimetic bnAbs and shares nine somatic mutation-encoded amino acids with VH1-46 class bnAbs that likely contribute to improved Env binding. To analyze how well the immunogen binds orthologous bnAb precursors, the authors identified additional sequences of the DH1285 clone and inferred a UCA. This DH1285 UCA displayed an eightfold weaker binding affinity for the immunogen than CH235 UCA with fivefold lower on-rates. M5.G458Y Env can thus engage and expand Ab precursors even if they have slower on-rates and weaker binding affinity for the immunogen than the CH235 precursor it was engineered to bind. Binding affinity improved as the DH1285 clone acquired mutations over time and specific amino acid changes were correlated to increasing CH505 TF reactivity. Together, engaging the VH1-46 class CH235 bnAb lineage by this engineered HIV immunogen allowed selecting for similar BCRs in macaques and elicited a bnAb precursor with VH1-46-like properties.

This study provides important proof of concept for the rational design of vaccines that trigger VH1-46 HIV bnAbs and demonstrates the usefulness of rhesus macaques as model system for this vaccine class. These encouraging results open new lines of investigation in the global efforts to develop a protective HIV vaccine. While the current strategy already shows remarkable success, fine-tuning of adjuvants properties but also modified prime/boost immunization regimens including immunogens designed to stimulate somatic hypermutation to shape development of germline precursor into bnAbs likely bears potential for further optimization. With HVTN309 and HVTN312, the authors already prepare two clinical trials to test the potency of VH1-46-targeted immunogens in humans.^5^
